# Multi-scale X-ray computed tomography to detect and localize metal-based nanomaterials in lung tissues of *in vivo* exposed mice

**DOI:** 10.1038/s41598-018-21862-4

**Published:** 2018-03-13

**Authors:** Perrine Chaurand, Wei Liu, Daniel Borschneck, Clément Levard, Mélanie Auffan, Emmanuel Paul, Blanche Collin, Isabelle Kieffer, Sophie Lanone, Jérôme Rose, Jeanne Perrin

**Affiliations:** 1 Aix Marseille Univ, CNRS, IRD, INRA, Coll France, CEREGE, Aix-en-Provence, France; 2 Univ Avignon, Inst Mediterraneen Biodiversite & Ecol Marine & C, Aix Marseille Univ, CNRS, IRD, Marseille, France; 3 AP HM La Conception, CECOS, Lab Reprod Biol, Dept Gynecol Obstet & Reprod Med, Pole Femmes Parents Enfants, Marseille, France; 4 International Consortium for the Environmental Implications of Nanotechnology iCEINT, CNRS–Duke University, Aix en Provence, France; 5 INSERM, Equipe 04, U955, Creteil, France; 6 Univ Paris Est Creteil, IMRB, Fac Med, DHU A TVB, Creteil, France; 7 OSUG-FAME, UMS 832 CNRS-Univ. Grenoble Alpes, F-38041 Grenoble, France

## Abstract

In this methodological study, we demonstrated the relevance of 3D imaging performed at various scales for the *ex vivo* detection and location of cerium oxide nanomaterials (CeO_2_-NMs) in mouse lung. X-ray micro-computed tomography (micro-CT) with a voxel size from 14 µm to 1 µm (micro-CT) was combined with X-ray nano-computed tomography with a voxel size of 63 nm (nano-CT). An optimized protocol was proposed to facilitate the sample preparation, to minimize the experimental artifacts and to optimize the contrast of soft tissues exposed to metal-based nanomaterials (NMs). 3D imaging of the NMs biodistribution in lung tissues was consolidated by combining a vast variety of techniques in a correlative approach: histological observations, 2D chemical mapping and speciation analysis were performed for an unambiguous detection of NMs. This original methodological approach was developed following a worst-case scenario of exposure, i.e. high dose of exposure with administration via intra-tracheal instillation. Results highlighted both (i) the non-uniform distribution of CeO_2_-NMs within the entire lung lobe (using large field-of-view micro-CT) and (ii) the detection of CeO_2_-NMs down to the individual cell scale, e.g. macrophage scale (using nano-CT with a voxel size of 63 nm).

## Introduction

In the past years, nanotechnology has undergone rapid development because of the enhanced or modified properties (e.g. magnetic, electronic, optic, surface chemical reactivity, etc.) of nanomaterials (NMs) compared to their bulk counterpart. Among others, cerium oxide NMs (CeO_2_-NMs) gained interest in many different fields including industrial applications benefiting from their catalytic activities (semiconductor, fuel combustion) and applications in nanomedicine (novel therapeutics for cancer or Alzheimer’s disease) benefiting from their antioxidant activities^1^. Conversely, such properties can also induce toxic effects both *in vitro* and *in vivo*. Moreover, a recent study^2^ showed that NMs may exhibit different biological kinetics *in vivo* according to their size, coating or targeting. To go further in the understanding of the toxicity mechanisms *in vivo*, analytical developments are required to thoroughly study the biodistribution and biotransformation of the NMs in organisms, tissue and even biological cells.

Bio-imaging (*in vivo* or *ex vivo*, 2D or 3D) is an expanding field with the development of state-of-the art techniques such as electron microscopy (SEM, TEM)^3^, atomic force microscopy (AFM)^4^, confocal fluorescence microscopy^5^, or positron emission tomography/computed tomography^6^. To date, the challenge in nanotoxicology is to detect and locate small objects as NMs and NMs aggregates in soft organs, tissues and biological cells. This required the use of bio-imaging techniques with high spatial resolution and sufficient contrast. The highest resolution of biological structures is generally achieved using optical or transmission electron microscopy e.g.^3^, but requires destructive manipulation of the 3D tissue structures. Indeed this method entails extensive tissue processing steps, which are very time-consuming, laborious and irreversible: dehydration, chemical fixation, staining with exogenous contrast agents, resin-embedding and precise slicing into thin sections. These manipulations are prone to many artifacts such as structural artifacts introduce by sectioning process (tissue shrinkage and deformation) and loss of data as a consequence of mechanical damage of fragile section^7^. In the case of studying the internalization of NMs in organs or tissues, the use of sectioning techniques can potentially led to the sample damage across the structure of interest and to the displacement of the NMs in the tissues. The quality of the results and the data interpretation are then strongly sample preparation-dependent.

To get around those difficulties, non-invasive 3D imaging techniques are preferable to visualize internal structure of intact biological samples. Such well-established (e.g. confocal microscopy) and emerging techniques (e.g. optical projection tomography or magnetic resonance microscopy)^8,9^ exist but exhibit own limitations. For example, optical projection tomography can only be applied to transparent sample and its achievable resolution is limited to the order of a few microns^10^. The choice of the most appropriate technique is therefore a question of compromise^11^. X-ray micro-computed tomography (micro-CT) and its recent substantial advances in term of spatial resolution, can overcome such limitations. This technique can readily resolve micron-scale features in opaque sample with dimensions measured in mm to cm.

Most of the early applications of X-ray computed tomography (CT, in the 1970’s) were for medical imaging. Nowadays micro-CT has become well-established for imaging diverse mineralized tissues (e.g. osteo and dental microstructure)^12^ or the anatomy of a variety of organisms (e.g. insects, vertebrates, invertebrates^13–16^. It has also been used for developmental studies (e.g. morphology of chicken embryos)^17^. Micro-CT analyses of soft tissues (e.g. organs) are more difficult because of the low intrinsic X-ray contrast of non-mineralized tissues^18^ but can thoroughly reveal the structure of organs^14,19^. For example, micro-CT coupled with specific breath-hold techniques, can image the lung *in vivo*^18,20–22^ with resolution of 30 µm. Micro-CT also allows the visualization of small pulmonary structures (e.g. bronchiole, alveolar duct) and alveolar architecture in *ex vivo* fixed lung, with resolution of 1–2 µm^21,23–25^.

The use of micro-CT to detect and locate NMs within soft tissues was gaining interest (e.g.)^26,27^ especially since the scientific community cautiously addresses the potential risks of NMs for humans and living organisms^28^. Particular interest has focused on the distribution of NMs in pulmonary tissue as the breathing is considered to be one of the main routes of NMs uptake by humans^27,29,30^. As an example, micro-CT (with a voxel size of 7–9 µm) has recently been used in a toxicological study to visualize the pulmonary distribution of iron oxide-coated polystyrene NMs after acute exposure in an *ex vivo* rabbit lung model^29^. In another recent study^27^, the bio-persistence of inorganic nanotubes (Ge-imogolites) and their biodistribution in rat lung were assessed by *ex vivo* micro-CT analyses (with a voxel size of 4.2 µm) after *in vivo* exposure. In these studies, the detection of NMs was restricted by the spatial resolution of the micro-CT that only allows the detection of NMs aggregates larger than the voxel size. Indeed single NM and aggregates smaller than the voxel size were not detected.

Micro-CT has tremendously evolved over the past decade with much more sensitive detection systems and increased spatial resolution. Recent micro-CT systems were designed to perform multi-scale analysis without reducing sample size (called local tomography). The advantage of such systems is to combine pre-visualization of the sample at low resolution with high-resolution imaging of selected region of interest^21,31^. Even more interesting in the case of nanotoxicology, it is now possible to reach spatial resolution of tens of nanometers to image cellular and subcellular structures^32–36^. These promising developments, initiated on synchrotron beamlines^37^, are now available at laboratory scale^31,35,38–40^.

The objective of this study was to develop an original methodology for the *ex vivo* detection and 3D location of NMs within soft tissues. This approach was developed following a worst-case scenario of exposure, i.e. mice were exposed *in vivo* by intra-tracheal administration to a high dose of CeO_2_-NMs. The originality of the methodology was to combine 3D imaging both at the micro-scale, using micro-CT and at the nano-scale, using innovative lab-bench X-ray nano-computed tomography (nano-CT) system. Moreover, a correlative framework^41^ based on chemical mapping, speciation analysis and histological observations for the same region of interest was proposed to consolidate the methodology for an unambiguous detection of metal-based NMs within the lung lobe.

## Results

### Ce quantification in lung tissue: total concentration and chemical mapping

Mice were exposed by intra-tracheal instillation (25 µL) to a single dose of 50 µg CeO_2_-NMs. One week after administration, mice were sacrificed and the total concentration of Ce in exposed lung (right lobes of the 5 exposed mouse were pooled) was quantified by inductively coupled plasma mass spectrometry (ICP-MS) at 450 µg of Ce/g of dried lung (with an experimental error of 0.2%).

**Figure 1:**
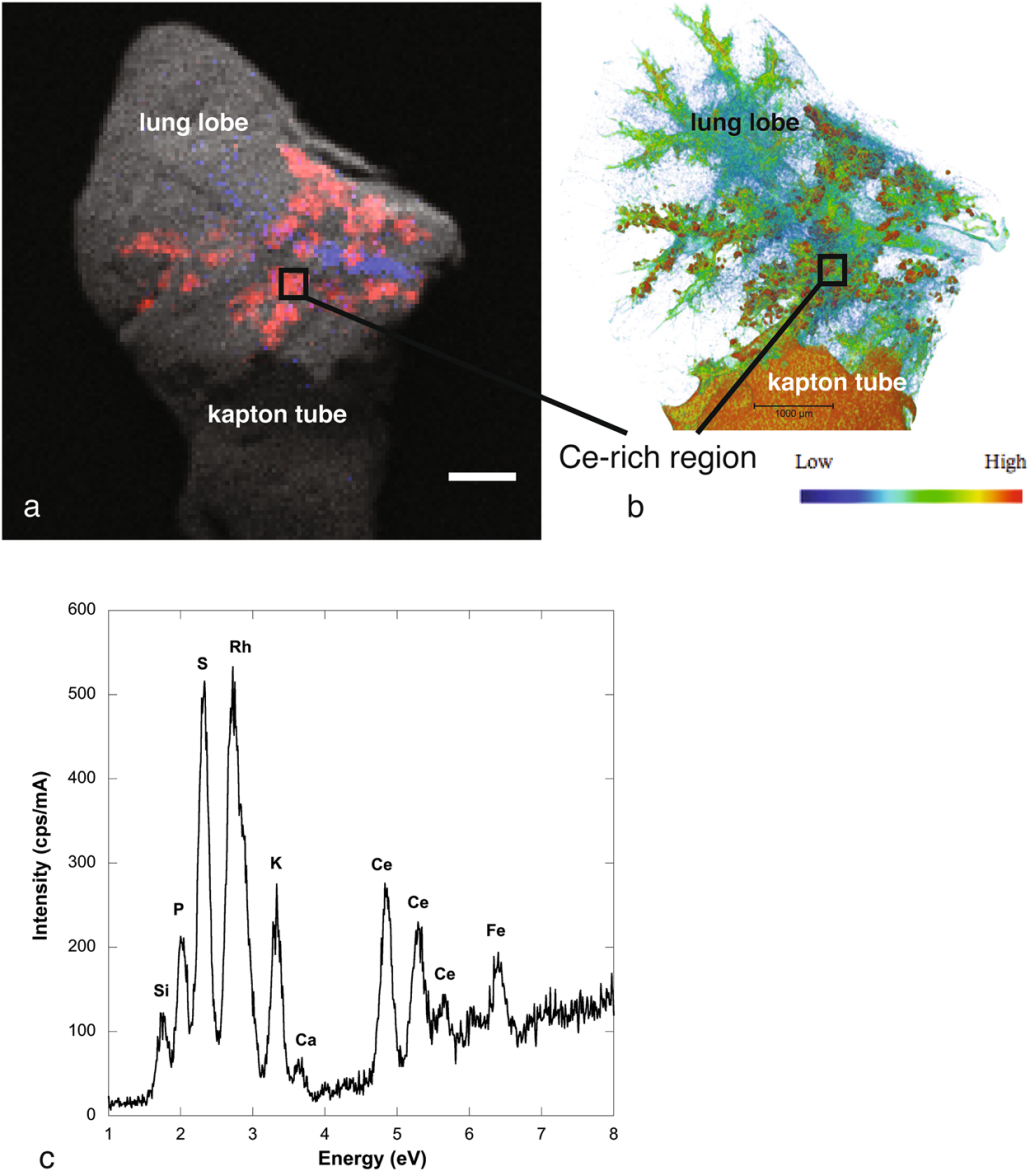
(**a**) 2D chemical mapping of critical point dried exposed lung by micro-XRF (1 px = 60 µm, scale bar = 1000 µm). Elemental maps of S (gray pixels), Fe (blue pixels) and Ce (red pixels) are combined in a tricolor map. (**b**) Volume rendering of micro-CT reconstructed image of the same sample (LFOV scan, 1 vx = 14.32 µm). The color map, from blue to red, indicates the X-ray attenuation (**c**) Average XRF spectrum extracted from the micro-XRF hyperspectral map (Ce-rich region labeled with a black frame on images (**a**) and (**b**)).

2D chemical mapping of a lung lobe by micro X-ray fluorescence spectroscopy (micro-XRF) (S, Fe and Ce distribution, Fig. 1a), revealed that the distribution of Ce in the lung lobe of exposed mice was non-uniform. Except Fe signal attributed to the presence of residual blood in the sample, Ce was the element with the highest atomic number (Z) detected in the lung (Fig. 1c). It is noteworthy that the Ce concentration in control sample (non-exposed mice) was below the ICP-MS limit of quantification and the micro-XRF limit of detection. Consequently detection of Ce using both ICP-MS and micro-XRF only originates from the CeO_2_-NMs initial administration.

### Chemical speciation of Ce detected in exposed lung tissue

As mentionned previously, Ce was detected by chemical analysis in exposed lung lobe, suggesting the presence of CeO_2_-NMs in lung tissue. But as initial CeO_2_-NMs can be subjected to biotransformation (e.g. partial reductive dissolution of Ce^4+^ to Ce^3+^) when reaching biological media (e.g.)^42^, *in-situ* Ce speciation analyses were required. These analyses were performed by X-ray Absorption Near Edge Structure (XANES) measurements to obtain information on the site geometry and on the electronic structure of the probed element (i.e. Ce). Moreover, the fluorescence detection at very high-energy resolution (HERFD) offered more defined edge and pre-edge features for analysis^43^.

**Figure 2:**
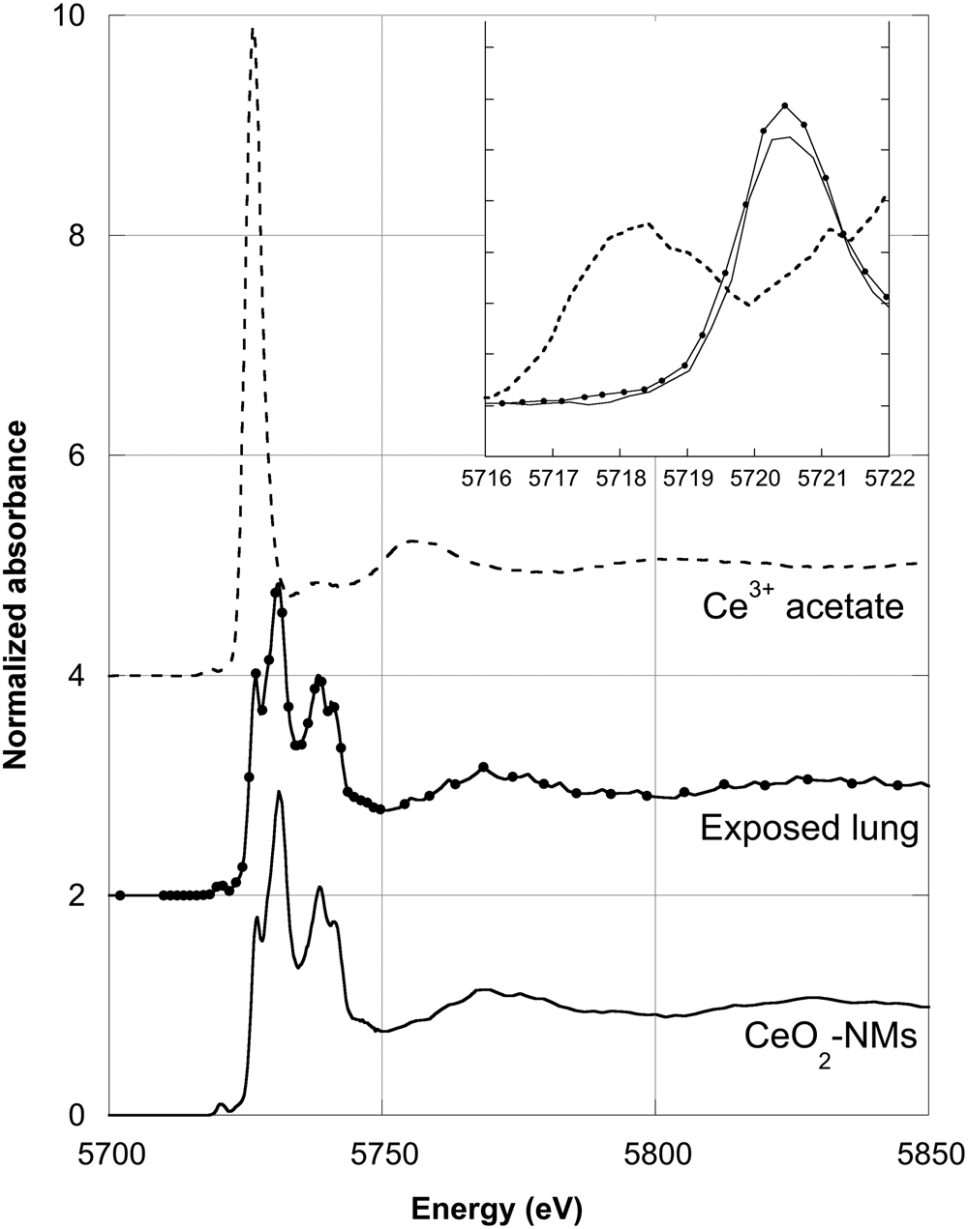
Ce L_3_-edge High Energy Resolution Fluorescence Detected (HERFD) X-ray absorption near-edge structure (XANES) spectrum of Ce detected in the exposed lung lobe. This spectrum is compared to initial CeO_2_-NMs (Ce^4+^) and Ce^3+^ acetate reference spectra.

In Fig. 2, HERFD-XANES spectrum of exposed lung (recorded at Ce L_3_-edge on CRG FAME/UHD beamline, ESRF, France) was compared to initial CeO_2_-NMs spectrum. The two spectra exhibited the same structures (edge, pre-edge, etc.), specifically no energy shift was observed on the pre-edge peak position. Then HERFD-XANES measurements confirmed unambiguously the presence of non-transformed CeO_2_-NMs in exposed lung tissue. Detected Ce can then be attributed to the presence of CeO_2_-NMs.

### 3D imaging of the lung tissues by micro-CT

**Figure 3:**
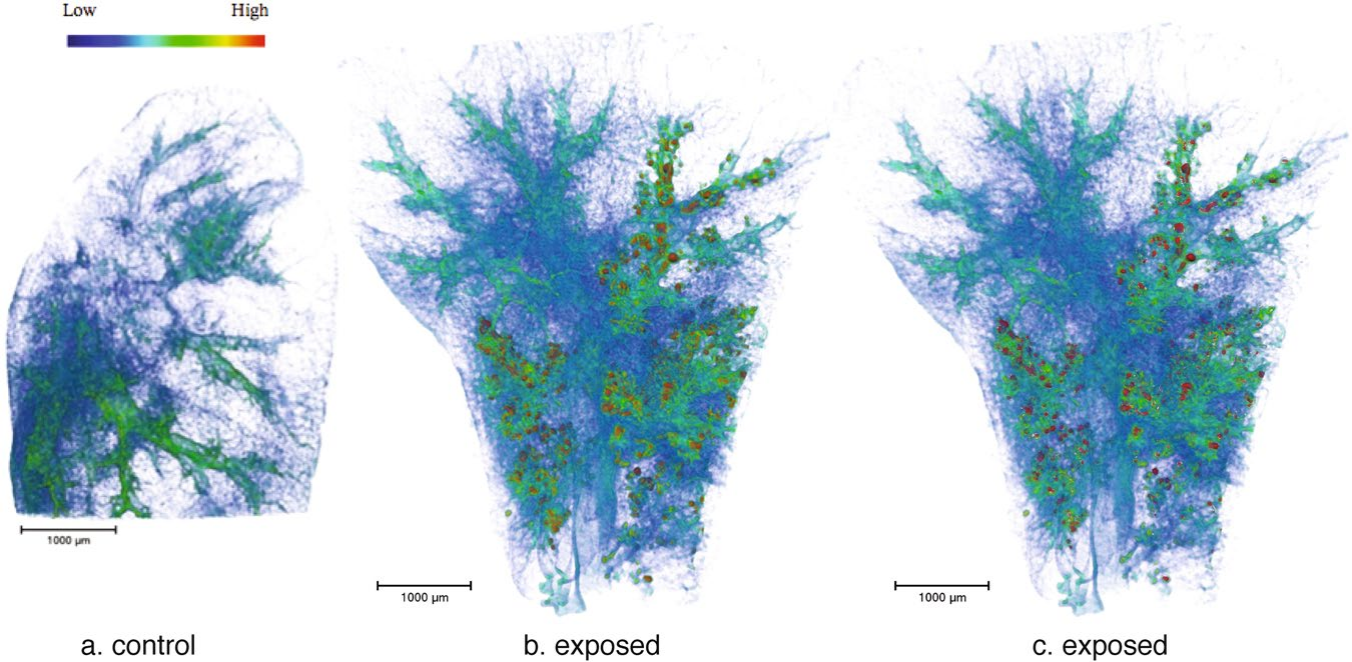
Volume renderings of micro-CT reconstructed images of lung tissues (LFOV scan, 1 vx = 14.32 µm: (**a**) control and (**b**) exposed samples. (**c**) Segmented voxels (voxels of (b) denser than threshold value and assigned to NMs) are colored in red (constant color). Sample holder (Kapton tube and glue) was removed from the images. The colormap, from blue to red, indicates X-ray attenuation.

Figure 3 shows volume renderings of control (5.A) and exposed (5.B) lung lobes obtained by micro-CT with a voxel size of 14.32 µm (Large Field Of View (LFOV) scan).

Normalization of the histogram and 3D image thresholding (procedure detailed in Methods part) have enabled the detection and isolation of the most brilliant voxels in the exposed lobe, denser than the denser voxel in the control lung tissue (red voxels in Fig. 3c). CeO_2_-NMs exhibit a high density and consequently a high X-ray mass attenuation coefficient, especially as compared to lung tissue and blood (4.293; 0.269 and 0.271 cm^2^/g at 40 keV respectively, from NIST database). We therefore assumed that the most brilliant voxels revealed the location of CeO_2_-NMs. Then the use of 2D chemical mapping and speciation analysis in combination validated this hypothesis and the thresholding procedure applied to isolate and locate voxels attributed to NMs. Indeed the location of the most brilliant voxels was in good agreement with the Ce distribution observed on the same sample by micro-XRF (Fig. 1a).

**Figure 4:**
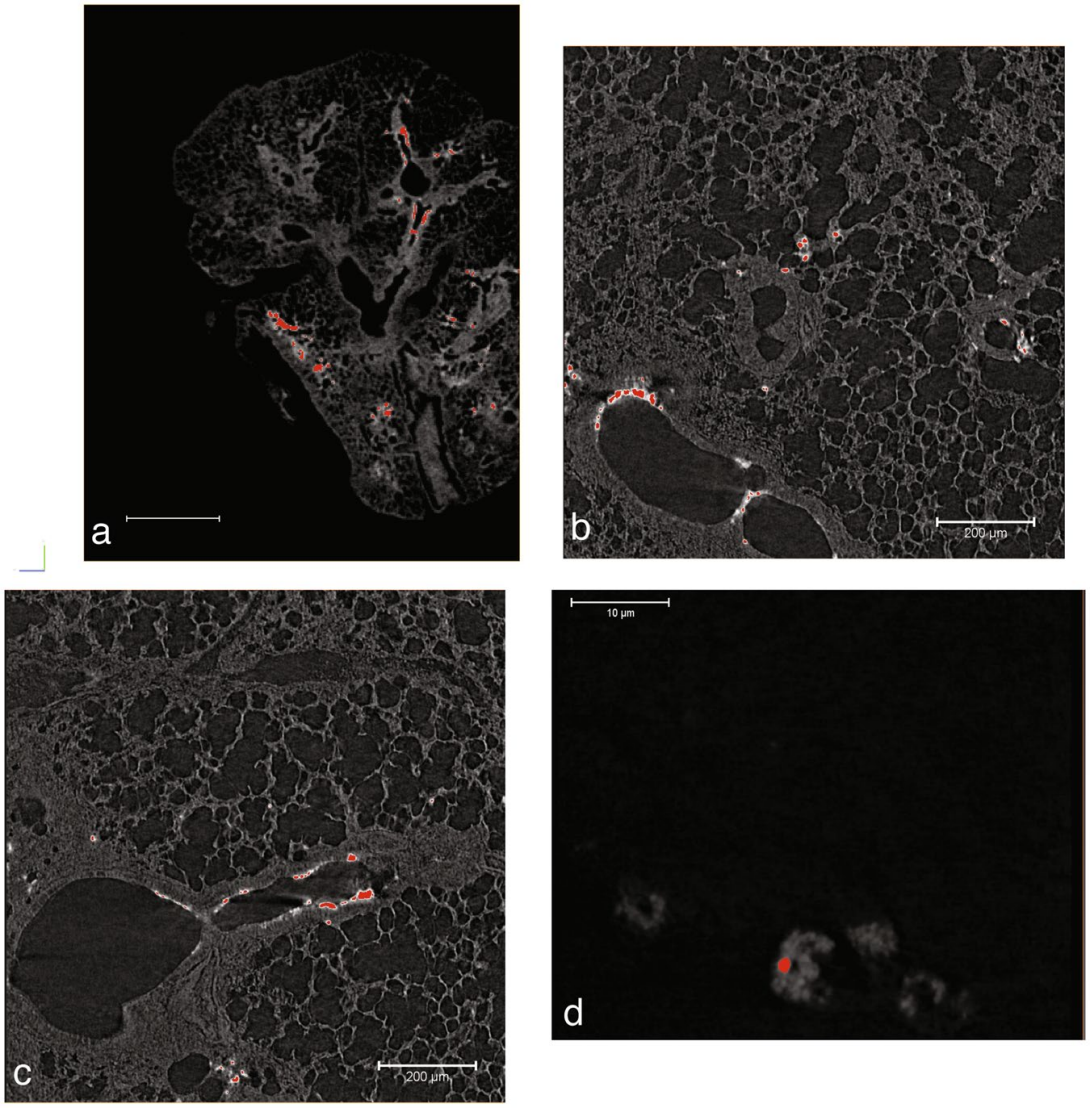
2D slices virtually extracted from reconstructed 3D images, a grayscale colormap is used. Segmented pixels are colored in red. (**a**) LFOV scan, 1 px = 14.32 µm, scale bar = 1000 µm (**b**,**c**) HRes scan, 1 px = 1.09 µm, scale bar = 200 µm (**d**) nano-CT scan, 1 px = 63.5 nm, scale bar = 10 µm.

Micro-CT results revealed that the 3D distribution of CeO_2_-NMs in the lung lobe, following intratracheal instillation, was non-uniform (Fig. 3c). With a voxel size of 14.32 µm (LFOV scan), large NMs accumulation regions were detected in the conducting and respiratory airway as well as in the alveolar parenchyma of mice exposed to CeO_2_-NMs (Figs 3c and 4a). These regions exhibited a mean equivalent circular diameter of 52 ± 20 µm, with a maximum size of 150 µm and a minimum size of 30 µm. It should be noted that accumulation regions smaller than 2 voxels (i.e. <28 µm for LFOV scan) couldn’t be reasonably quantified^44^. By increasing spatial resolution (HRes scan with a voxel size of 1.09 µm), smaller NMs accumulation regions were detected at the same location (i.e. parenchyma and airways) (Fig. 4b and c). Their size ranged from 2.18 to 42 µm with an average equivalent circular diameter of 7 ± 4 µm.

### Analysis of CeO_2_-NMs accumulation region at high spatial resolution: 3D imaging by nano-CT and 2D histological observations

**Figure 5:**
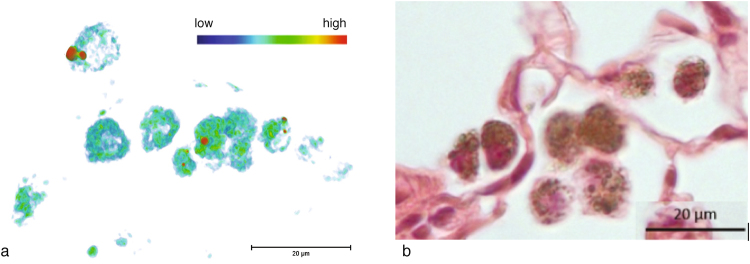
Macrophages with dense cytoplasm observed in exposed lung lobe. (**a**) *In-situ* and in 3D in critical point dried sample (nano-CT scan, 1 vx = 63.5 nm, color map indicates X-ray attenuation, red voxels are assigned to NMs by thresholding procedure). (**b**) In 2D from histological observations (x40) in paraffin-embedded and stained lung tissue section. Scale bars = 20 µm.

Figure 5a shows the reconstructed 3D image obtained by nano-CT with the field of view (FOV) centered on CeO_2_-NMs accumulation region identified in exposed lung lobe by LFOV micro-CT (Figure S1 in Supporting Information). The circular structures observed on nano-CT images (Figs 5a and 4d) can be attributed to macrophages with dense cytoplasm. Indeed shape and size of these objects are similar to macrophages observed by histology in Ce-rich region of exposed sample (Figs 5b and S2d). Histological observations revealed the presence of dense particles (black pixels) in the cytoplasm of macrophage cells located in the parenchyma alveolar walls. In control sample, these macrophages with dense cytoplasm were not observed (Figure S3). Reconstructed nano-CT image of the dense macrophages (Fig 5a) shows clearly the presence of very dense voxels in their cytoplasm. By using the thresholding procedure described in the Methods section (Figure S4), these voxels can be identified as CeO_2_-NMs accumulation regions and be isolated. Analyze of binary image obtained by thresholding and quantification of each isolated objects (assimilated to individual CeO_2_-NMs aggregates) revealed that CeO_2_-NMs aggregates detected in the cytoplasm of dense macrophages had a size between 300 to 2373 nm.

## Discussion

This study proposes a consolidated approach that combines several tools and benefits from recent technical developments to monitor the biodistribution of metal-based NMs within a lung lobe. Our approach is ambitious in being the most effective for *ex vivo* 3D imaging after *in vivo* exposure. It is noteworthy that this study was not designed to evaluate the toxicological impact on lung following a realistic CeO_2_-NMs exposure.

First, our methodology relies on an optimal sample preparation for 3D imaging: i.e. a simple, fast, non-destructive and artifact-free preparation of the sample. Briefly, the lobe was fixed in formalin, soaked in ethanol and subjected to critical point drying. Micro-CT was routinely used for the investigation of the internal anatomy and morphology of organisms while biological soft tissues produce low X-ray absorption contrast. In order to enhance the contrast, the most common and easiest method consisted in staining the specimen, i.e. labeling the structure of interest with high Z-elements probes (such as osmium, gold, iodine, barium…)^13,14,17,18,25,45,46^. However chemical staining is not suitable for dense metal detection (dissolved or nanoparticulate forms) as the labeling staining agent can potentially mask their presence. Recent advances on sample preparation techniques, e.g. ethanol fixation^47^, paraffin embedding^35^ or critical-point-drying^48,49^ can overcome this difficulty. Critical point drying enhances contrast by removing water that has similar X-ray absorption coefficient to many soft tissue components^48^. This drying process was commonly used to preserve the structure of biological samples for scanning electron microscopy^50^ as it minimizes artifacts such as shrinkage of tissue and distortion. It was then adapted to dry lung tissues with relatively high inherent contrast compared to other biological tissue^18^. Moreover, the sample preparation procedure developed in this study was relatively low-time consuming compared to other procedure, e.g. the fixation method proposed by Vasilescu *et al*.^51^.

Secondly, our methodology relies on recent improvements of nano-CT system and on the opportunity to perform multi-scale imaging up to the nano-scale^21,35^. In 2012, Vasilescu *et al*.^21^ demonstrated that micro-CT, with a voxel size of 2 µm, provided a very precise structural imaging of the pulmonary acini, with qualitative and quantitative description of these structures. In the context of metal-based NMs biodistribution, such a voxel size of 2 µm is not sufficient. Indeed precise and efficient visualization of NMs within biological structures requires imaging at very high spatial resolution, i.e. up to the nano-scale. In this study, we used a combination of two lab-bench CT systems (micro and nano-CT) to locate metal-based NMs in mouse lung tissues down to the cellular scale, with a voxel size ranging from 14.32 µm to 63.5 nm. Advantage of this multi-scale approach is to combine pre-visualization of the whole organ at low resolution with high-resolution imaging of selected volume of interest on the same sample.

The LFOV micro-CT images revealed the non-uniform accumulation of CeO_2_-NMs aggregates in the conducting airways (bronchial region) and in the distal zone of the lung (alveolar parenchyma) (Figs 3c and 4a). This non-uniform distribution, at the lobe scale, was expected after NMs intra-tracheal instillation^52^. At such low spatial resolution, only larger NMs accumulation regions (with equivalent diameter ranging from 30 to 150 µm) were observed. Reducing voxel size, i.e. increasing spatial resolution, is then required to provide complementary data of the fine location at the tissue and cellular scale. At higher spatial resolution (HRes micro-CT scan with a voxel size of 1.09 µm), smaller NMs accumulation regions (with equivalent diameter ranging from 2 to 42 µm) were detected in the parenchyma (Fig. 4b and c). Such accumulation regions were not observable in LFOV micro-CT images because they exhibit similar or smaller size compared to the voxel size. By increasing further the resolution up to the nano-scale, NMs accumulation regions, with equivalent diameter ranging from 300 to 2373 nm were identified inside the cytoplasm of macrophages in the alveolar wall of the parenchyma and more precisely in endocytosis vesicles (Figs 4d and 5). It should be noted that even using nano-CT, the spatial resolution remains too low to locate individual CeO_2_-NMs.

These results on the CeO_2_-NMs distribution within lung tissue can be compared with two other studies using *ex viv*o micro-CT. In the study published by van den Brule *et al*.^27^, aggregates of nanotubes (62 or 70 nm length) were localized by micro-CT (voxel size of 4.24 µm) in the lung dense fibrotic alveolar areas of rats exposed *in vivo* by intratracheal instillation. Beck-Broichsitter *et al*.^29^ focused on the biodistribution of polystyrene-coated iron oxide NMs in an isolated, ventilated and perfused rabbit lung model (IPL) after an acute *ex vivo* exposure. They detected NMs aggregates only in the conducting and respiratory airways by micro-CT (voxel size 7–9 µm). In these studies, detection of individual NMs and smaller aggregates (smaller than voxel size), potentially dispersed in the parenchyma, was restricted by the spatial resolution of micro-CT limited to the micro-scale. Moreover, it is noteworthy that results should be carefully compared since experimental conditions could also affect the NMs distribution, e.g. design of the isolated *ex vivo* lung with no pulmonary clearance and structure, chemical reactivity and size of the NMs.

**Figure 6:**
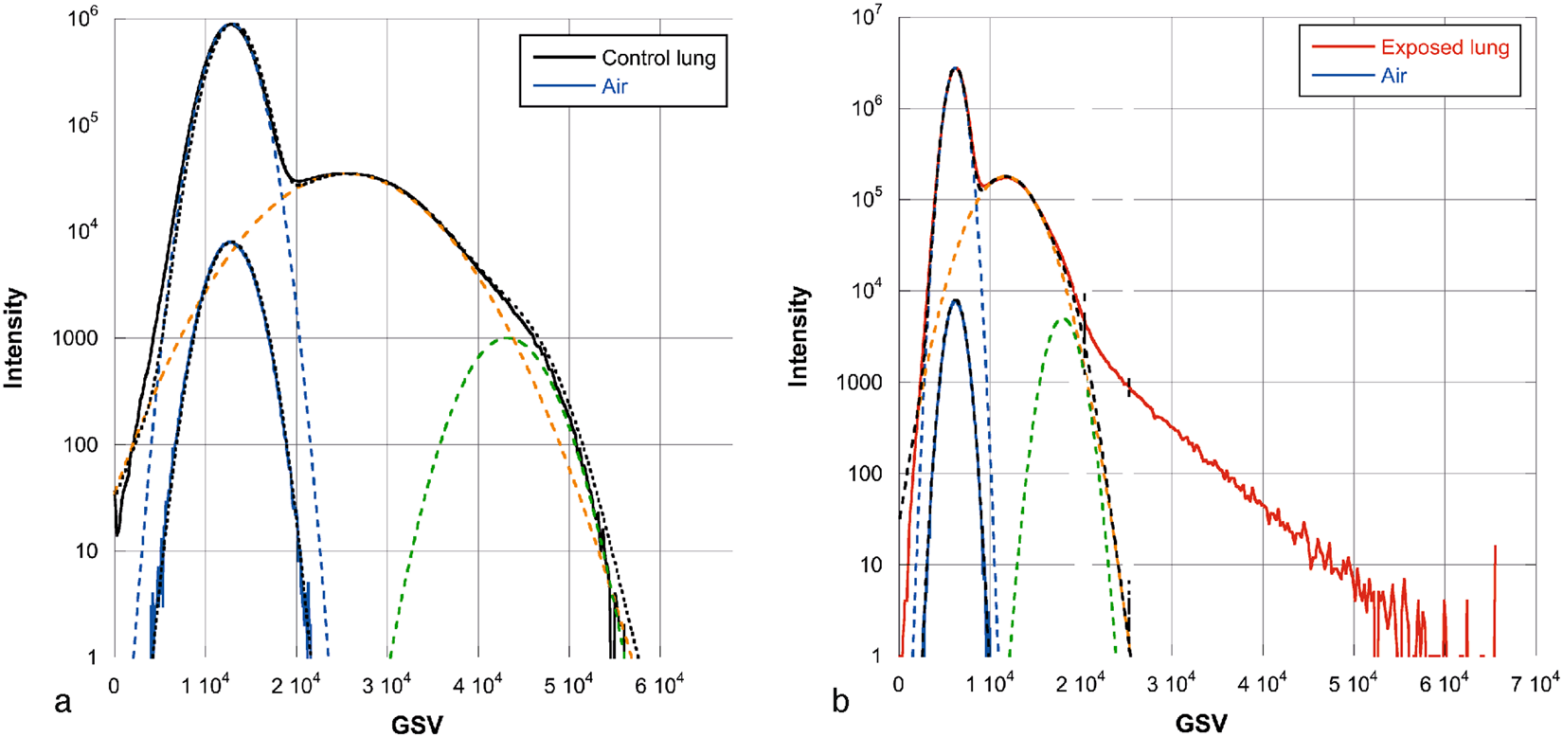
Histograms of sub-volumes extracted from micro-CT reconstructed images (LFOV scan, 1 vx = 14.32 µm): (**a**) control and (**b**) exposed samples. Sub-volumes of lung tissue (excluding sample holder, i.e. kapton tube) and sub-volume of air voxels are considered. First parts of lung tissue histograms are well fitted by the sum of 3 Gaussian functions (blue, orange and green dotted lines, black dotted lines are sum fit). Air sub-volume histograms are well fitted with a Gaussian function (black dotted line).

**Figure 7:**
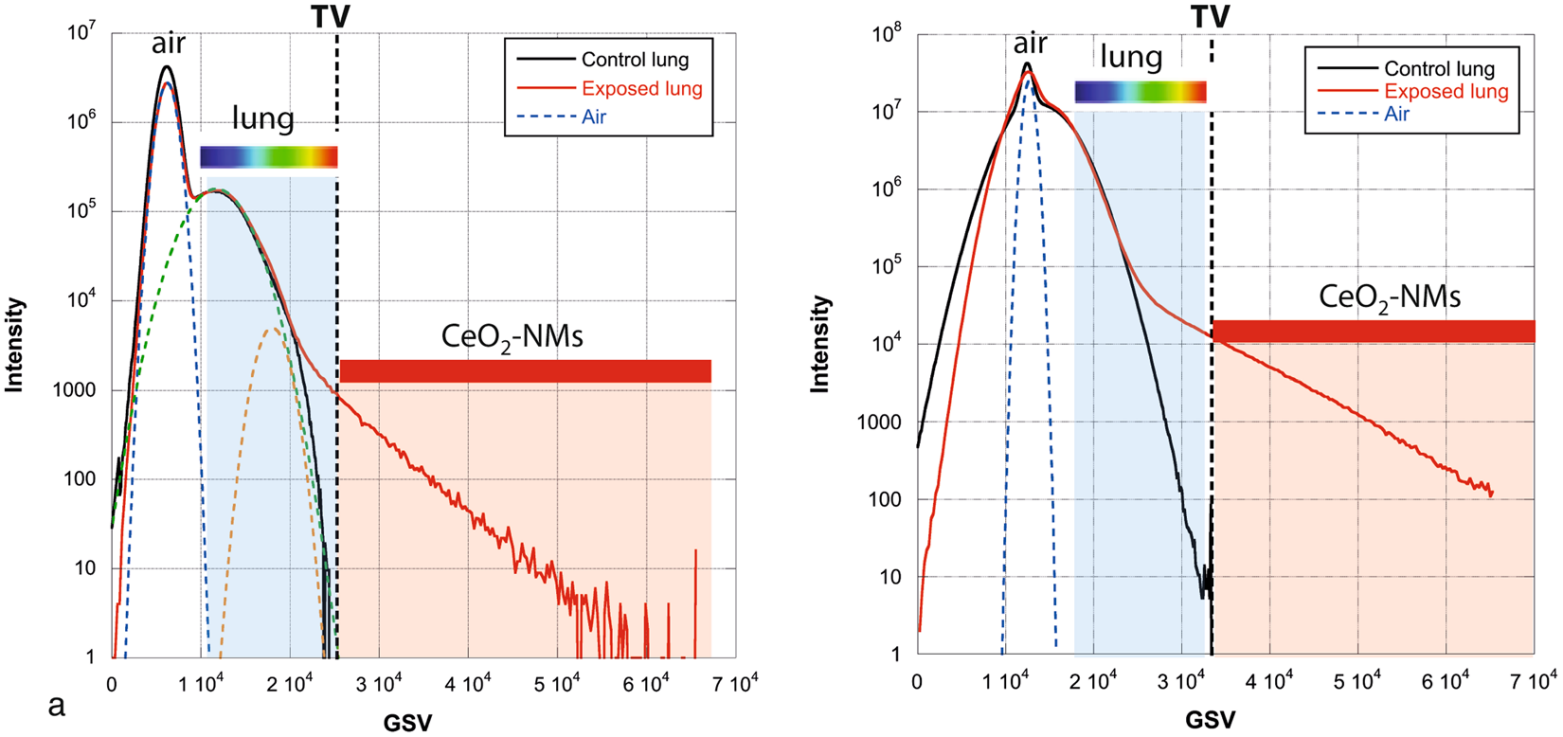
GSV-normalized histograms of lung tissue sub-volumes extracted from reconstructed images of control and exposed samples (**a**) LFOV and (**b**) HRes micro-CT scans. Voxels denser than threshold value (TV, dotted line) attributed to NMs, are colored in red in Figs 3c and 4.

Our study presentes a complete and original methodology to identify the biodistribution of metal-based NMs in the lung lobe. The methodology was mainly based on innovative and multi-scale 3D imaging coupling micro-CT and nano-CT analysis. As micro and nano-CT provides no direct chemical and speciation information, detection and location of NMs in 3D images were obtained following a multi-steps data analyze procedure: (i) histogram normalization; (ii) comparison of exposed and control sample and (iii) denser voxels thresholding (Figs 6 and 7). To consolidate NMs detection, 2D chemical mapping and speciation analysis were performed on the same sample (Figs 1 and 2). The good spatial correlation between the Ce-rich pixels in 2D chemical maps and the voxels of micro-CT images attributed to CeO_2_-NMs provided a good validation of the CT data analysis procedure. Then coupling nano-CT together with histological observation was a powerful and robust approach to obtain very precise information on CeO_2_-NMs distribution (Figs 5 and S2). Histological observations provide good quality images at high resolution of the lung tissue and its architecture as for example the macrophages located in the alveolar walls of the parenchyma (Fig. 5b). It should be noted that, as micro and nano-CT, this technique does not allow direct unambiguous detection of CeO_2_-NMs as no chemical information is given. To overcome this limitation, chemical mapping by micro-XRF was also used to identify Ce-rich regions specifically selected for light microscopy examination (Figure S2). then the dense particles observed in the cytoplasm of macrophage cells by histology can be identified as CeO_2_-NMs.

Even if histological observations are generally performed on multiple samples and multiple FOVs for a better representativeness, it could become tricky to find metal-rich or NMs-rich regions in a whole lung lobe or organ. Indeed, histological observations are generally performed on small areas selected on very thin sample slices. Combining histological observation with multi-scale 3D imaging (micro-CT and nano-CT) is then an interesting approach as 3D imaging have a larger FOV, is non destructive and does not exhibit artifact induced by sample slicing.

To conclude, the methodology developed in this study relied on recent technical advances of 3D X-ray imaging, was consolidated by a correlative approach that successfully provided precise information on metal-based NMs biodistribution, from the lobe scale to the cellular scale (e.g. macrophage). In the future, this methodology requires to be tested with more realistic exposure conditions (dose, route and timing of NMs administration). It also requires to be supplemented by a more statistical approach, based on analyses of various lobes, i.e. basal and upper part of the lung, in order to provide complementary information on NMs distribution at the lung scale. After such a validation, it could be applied in nanotoxicological studies and will represent a significant advance to precisely describe the metal-based NMs exposure related to toxicity results. This will help identifying the toxicity mechanisms following metal-based NMs exposure.

## Methods

### Synthesis and physical-chemical properties of CeO2-NMs

Bare CeO_2_-NMs (Nanograin®, Umicore) were suspended in ultrapure water at 10 g.L^−1^. They were crystallites of cerianite (identified by X-ray diffraction, XRD) with a transmission electron microscopy (TEM) size of 31 ± 18 nm (Figure S5). In their stock suspension (pH = 3.1), their average hydrodynamic diameter was centered on 90 nm with a zeta potential of 42 ± 2 mV. Their isoelectric point (IEP) was measured at pH~7.8 ± 0.2. Their specific surface areas (SSA) were estimated at 56 ± 10 m^2^.g^−1^.

### Animal experiments

The institutional Ethic Committee (C2EA-16, ComEth ANSES/ENVA/UPEC) approved the exposure protocol. All experiments were performed in accordance with relevant guidelines and regulations. C57/Bl6 mice (6–8 weeks old) were housed in a temperature and light-controlled room, with free access to food and water. Mice were either exposed to a single dose of 50 µg CeO_2_-NMs (5 exposed mice), or to the vehicle (water) (5 control mice) by intratracheal instillation (25 µl). Instillation with NMs was performed from stock suspension of CeO_2_-NMs after 15 min of ultra-sonication. This dose was used as representative of high occupational exposure as seen in welders^53^. The purpose of animal *in vivo* exposure was to provide lung samples containing metal-based NMs at relevant concentrations to be located by micro and nano-CT. Mice were sacrificed one week after administration. At that time, mice were anesthetized and saline solution was directly injected into the right cardiac ventricle to flush the blood and the lungs were removed.

### Sample preparation

Left lungs were inflated with 10% pre-filtered Formalin at a pressure of 20 cm H_2_O using a 22-gauge catheter and were fixed overnight at room temperature. After fixation, tissues were dehydrated in a graded series of ethanol and isopropanol solution and embedded in paraffin. Five micrometers thick medial frontal sections of lungs were stained with hematoxylin and eosin (H&E) for histological observations.

Right lungs (3 right lobes by mouse) were stored at −80 °C and subsequently used for chemical analysis (ICP-MS). The residual lobes (fourth right lobes) were dedicated to 2D and 3D imaging (micro-XRF, micro-CT and nano-CT) and to speciation analysis (HERFD-XANES). After fixation in formalin solution, lobes of exposed and control mice (approximately 5 × 3 × 3 mm) were soaked in successive ethanol solutions (from 30% vol. to 100% vol.) and critical point dried (Leica EM CPD300°). This drying process avoids the creation of damaging surface tension forces associated with drying by bringing liquid in the sample to the gas phase without crossing the liquid-gas phase boundary. Dried samples were finally glued (with epoxy glue) at the tip of a polyimide tubing (Kapton).

### Histology

The paraffin-embedded and stained lung tissue sections were observed using light microscopy (Axioplan 2, Oberkochen, Germany) with increasing magnification (from x5 to x40).

The base of the paraffin-embedded lobes sectioned for histological observations were scanned in 2D and 3D by micro-XRF and micro-CT (Figure S2a,b). Then the Ce-rich region identified was marked and selected for optical microscopy observation with increasing magnification (Figure S2c,d). The black frame on Figure S2 shows the position of the selected Ce-rich region.

### Elemental quantification of Ce by ICP-MS

Right lungs lobes of 5 mice by condition (exposed and control sample) were pooled. The pooled samples were dehydrated and then digested using a triacid mixture (5 mL HF, 5 mLHNO_3_ and 2 mL HClO_4_). Ce concentration was measured in lung tissues of exposed and control mice by ICP-MS (PerkinElmer, NexIon 300x). Sensitivity of ICP-MS for Ce detection is 3.10^−3^ µg/l. Results are expressed as µg of Ce/g of dried lung and represent mean value obtained from 5 mice.

### 2D chemical mapping by micro-XRF

Micro X-ray fluorescence spectroscopy (micro-XRF) was used to determine the elemental distribution of Ce, S and Fe in critical point dried lung lobe. 2D elemental maps were recorded in hyperspectral mode using a microscope (XGT-7000, HORIBA Jobin Yvon) equipped with an X-ray guide tube with a diameter of 100 µm (X-ray source with Rh target, accelerating voltage of 30 kV and current of 1 mA). Mapping of the whole lobe with pixel size of 60 µm was performed with a total counting time of 20 × 1000 s.

### Chemical speciation of Ce by HERFD-XANES

Cerium L_3_-edge (5723 eV) XANES measurements were performed on the CRG FAME/UHD (BM16) beamline at the ESRF (Grenoble, France) on exposed lung lobe. The specificity of this beamline is to carry out the measurements in fluorescence mode using a detection system (i.e. a crystal analyzer spectrometer, CAS) with very high-energy resolution ranged from 0.2 to 2 eV. The measurement is then called HERFD-XANES. The spectrometer was equipped with 5 spherically bent crystals Ge(331) in a Rowland geometry and a helium bag was used to limit the absorption of the fluorescence signal on the X-ray path from the sample to the crystals and to the detector. More details on the CAS and on the beamline are given in^43,54^.

Spectra acquisition was performed at liquid helium temperature to avoid sample evolution under beam. Spectrum obtained for exposed lung lobe was the sum of seven scans recorded with the incident beam pointed at two different positions. Data reduction was performed using an IFEFFIT software package^55^. Initial CeO_2_-NMs (Ce^4+^) and Ce acetate (Ce^3+^) were used as reference samples.

### 3D imaging by a combination of micro-CT and nano-CT: image acquisition

Dried samples (control and exposed samples) were first scanned in 3D using Zeiss XRadia Micro XCT-400 system. This instrument is designed to perform multi-scale micro-CT with isotropic voxels ranging from 50 µm down to 1 µm within a large sample without the need to section it. Voxel size is mainly defined by the combination of geometrical magnification (defined from source to sample and sample to detector distances) and optical magnification obtained with a turret of optical objectives combined with scintillators. The scintillators convert X-ray into visible light.

Micro-CT of the lungs was performed at two spatial resolutions: LFOV scan (MACRO objective), with an isotropic voxel size of 14.32 µm and a FOV of 14.66 × 14.66 × 14.66 mm^3^, containing the whole lobe and allowing selection of a region of interest for a high-resolution scan (HRes scan, 20x objective) with an isotropic voxel size of 1.09 µm and a FOV of 1.12 × 1.12 × 1.12 mm^3^. The FOV position of the HRes scan was centered in the parenchyma, outside from the largest NMs-rich accumulation regions in order to detect potential disperse and smaller NMs-rich regions.

Scans were acquired at 40 kV (W target) and 250 µA. During LFOV scan, 1201 projections were collected with an exposure time of 4 s per projection and an angle step of 0.30° (through the 360° rotation). During HRes scan, the number of projections and exposure time were increased to 2501 and 25 s, respectively, leading to an angle step of 0.144°.

Exposed sample was also scanned in 3D at the nano-scale using the Zeiss XRadia UltraXRM-L200 system equipped with a rotating anode X-ray source (Cu target, acceleration voltage of 40 kV, current of 30 mA) and Fresnel zone plate providing a spatial resolution of 150 nm. During the nano-CT scan, 901 projections were collected in absorption contrast mode during a 180° rotation of the sample and with an exposure time of 60 s/projection. The FOV was 65 × 65 × 65 µm^3^ with an isotropic voxel size of 63.5 nm.

The FOV position of the nano-CT scan (exposed sample) was selected from a mosaic image of 21 × 17 2D nano-CT raw projections (with a unit size of 65 × 65 µm and a pixel size of 63.5 nm, see more details in Figure S1).

Reconstruction of the volumes was done using a Zeiss XRadia software (XMReconstructed-Parallel beam-9.0.6445 software) using a Filtered Back Projection algorithm.

### 3D image treatment and analysis

Avizo 8.0 software was used for reconstructed dataset visualization, treatment and analysis. Detection and location of NMs within lung tissues are based on the comparison of the 3D images obtained for exposed and control samples. A first step of data normalization is required. In LFOV image, a sub-volume of the lobe, excluding kapton tubing and glue (sample holder) and a sub-volume containing exclusively air voxels were selected and their histograms were extracted. The histograms represent the X-ray attenuation in each voxel (expressed as an arbitrary Gray Scale Value, GSV) of the analyzed volume as function of the number of voxels for each GSV (intensity). The GSV depend on material composition (density) and thickness. Normalization of the histograms was performed using air as an internal standard and consisted in shifting and multiplying the histogram GSV axis by calculated factors so that the air contributions from the exposed and control samples (identified from air sub-volumes and well fitted with a Gaussian function) overlap (same maximum position and full width at half maximum) (Figs 6 and 7).

Histogram of control lung tissue sub-volume is well fitted by the sum of 3 Gaussian functions: a Gaussian function attributed to air contribution (with position and full width at half maximum FWHM determined from air sub-volume fit) and two additional Gaussian functions attributed to lung tissues contribution (voxels denser than air). After normalization, histograms of lung tissue sub-volumes for control and exposed sample can be superimposed and compared. The part of the exposed sample histogram (denser voxels at highest GSV) not fitted by air and lung tissue contributions is then attributed to CeO_2_-NMs by a segmentation step. A threshold value was set at the base of the third Gaussian function (Fig. 7) to identify the voxels associated to the NMs.

The same normalization and thresholding procedure was applied to 3D images obtained by micro-CT (HRes scan) (Figs 7b and S6) and nano-CT (Figure S4). It should be noted that the left part of the normalized histograms of HRes scans (control and exposed samples) did not overlap very well, this shift is due to reconstruction artifact^56^.

After thresholding, a labeling procedure was performed on obtained binary images to isolate and quantify each individual object, defined as CeO_2_-NMs accumulation regions. Equivalent circular diameter of each object was calculated.

### Ethics approval and consent to participate

The exposure protocol has been approved by the institutional Ethic Committee (C2EA-16, ComEth ANSES/ENVA/UPEC). All experiments were performed in accordance with relevant guidelines and regulations

### Availability of data and material

The datasets used and/or analysed during the current study are available from the corresponding author on reasonable request.

## Electronic supplementary material

Supplementary Information
