# Supplementary material for: Multi-scale X-ray computed tomography to detect and localize metal-based nanomaterials in lung tissues of in vivo exposed mice
Source: Sci Rep. 2018 Mar 13;8:4408. doi: 10.1038/s41598-018-21862-4 (PMC5849692; doi:10.1038/s41598-018-21862-4)
Supplement: Supplementary file 1 — Supplementary Information [file 41598_2018_21862_MOESM1_ESM.pdf]

Additional files

Multi-scale X-ray computed tomography to detect and localize metal-based nanomaterials in lung tissues of *in vivo* exposed mice.

Perrine Chaurand[1,4*], Wei Liu[1,4], Daniel Borschneck[1,4], Clément Levard[1,4], Mélanie Auffan[1,4], Emmanuel Paul[5,6], Blanche Collin[1,4], Isabelle Kieffer[7], Sophie Lanone[5,6], Jérôme Rose[1,4], Jeanne Perrin[1,2,3]

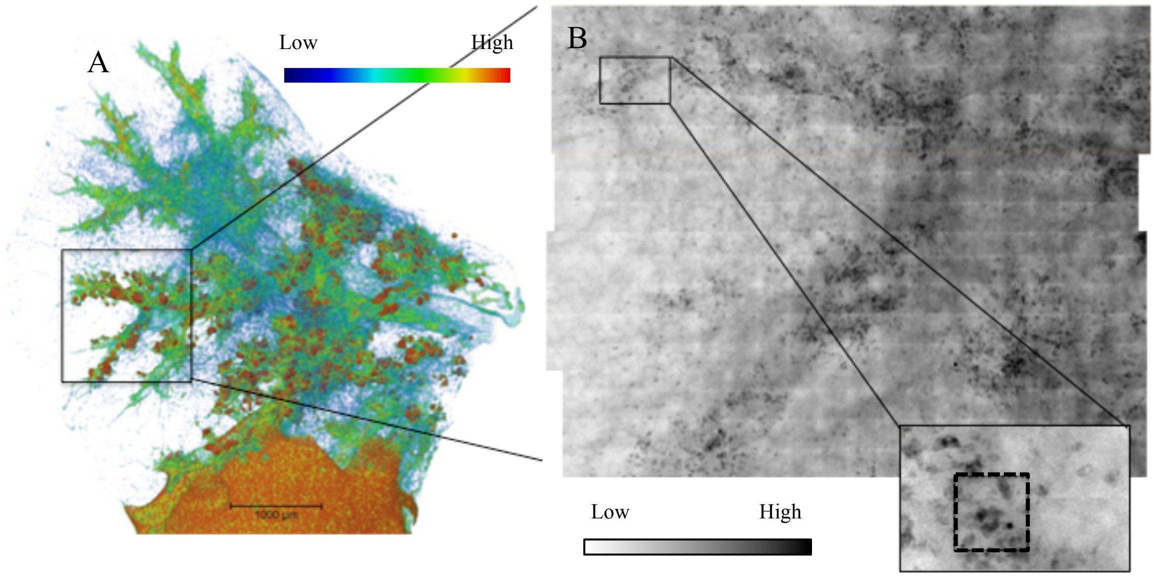

Figure S1: (A) Volume rendering of exposed sample (micro-CT, LFOV scan, 1 vx = 14.32 µm). The black frame indicates the region scanned in 2D by nano-CT. (B) Mosaic image composed of 21x17 2D nano-CT raw projections with a unit size of 65x65 µm and a pixel size of 63.5 nm. The dotted frame shows the FOV position of the nano-CT scan. Dense objects were observed (darker pixels in the raw 2D image) in CeO_2-NMs rich regions identified by micro-CT and are then suspected to contain NMs. The nano-CT FOV (dotted frame on the mosaic image, zoom) was centered on these dense objects.

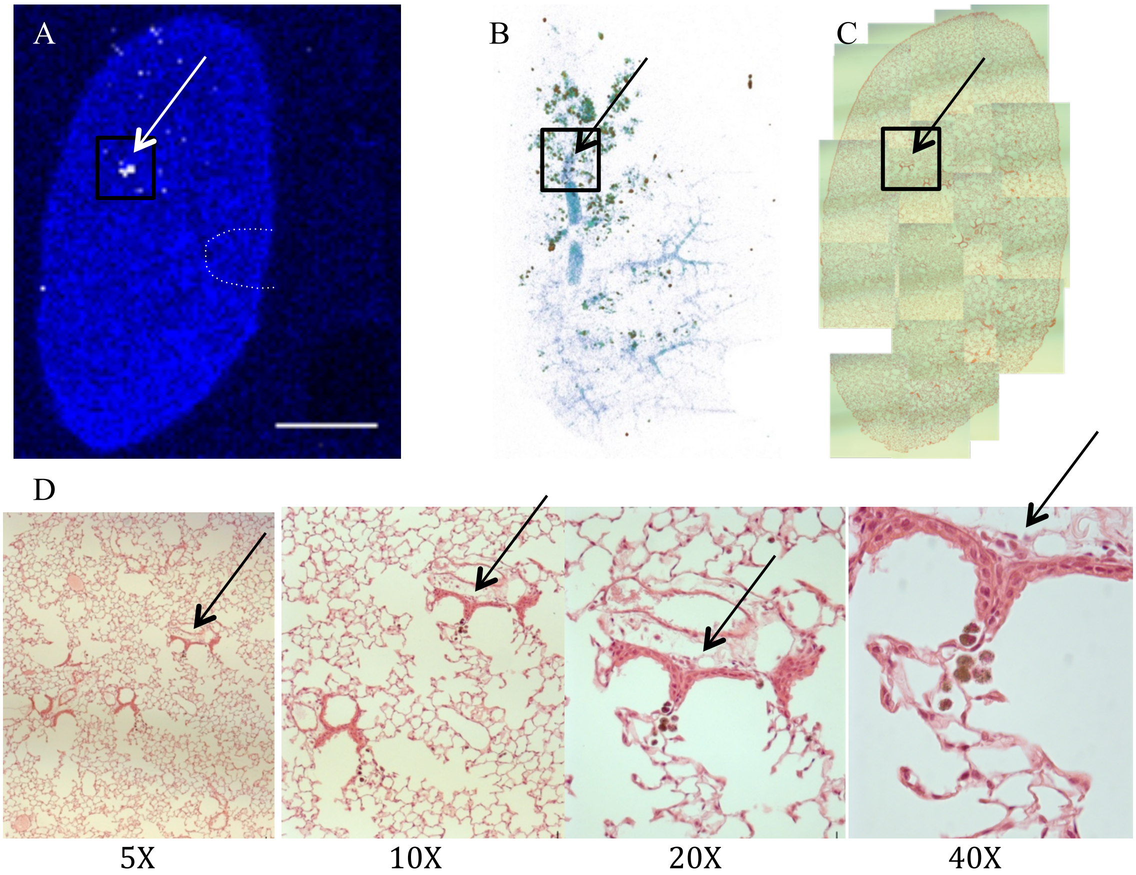

Figure S2: Exposed sample embedded in paraffin and sliced for (A) micro-XRF, (B) micro-CT and (C, D) histological observations of the same region. (A) 2D elemental maps of S (blue) and Ce (white) are combined (scale bar = 2 mm). Macrophages are observed by histological observations (x40) in the Ce-rich region (black square and arrow).

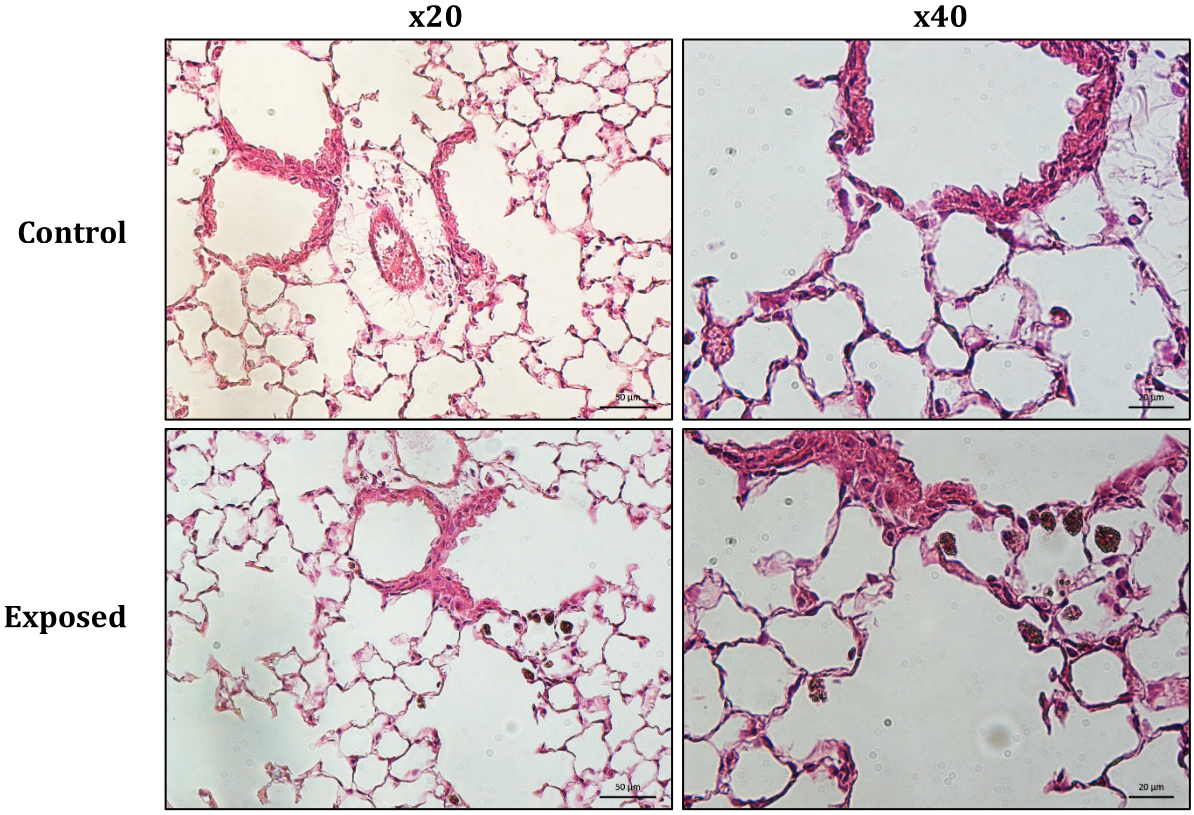

Figure S3: Histological observations of control and exposed samples with various magnification (x20 and x40).

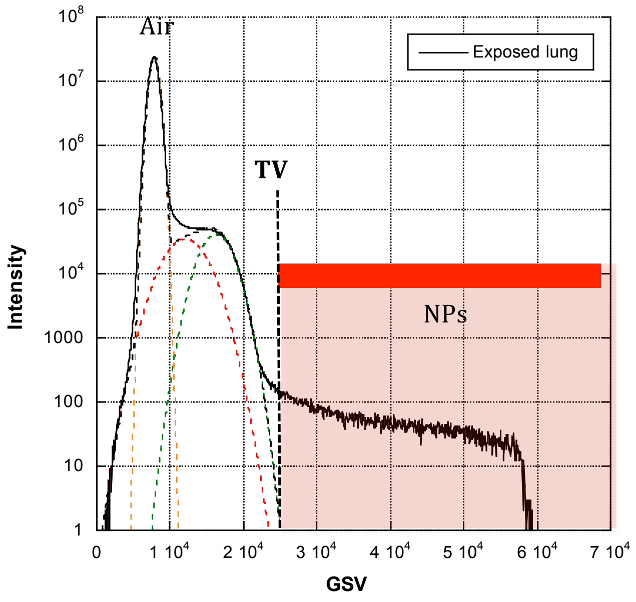

Figure S4: Histograms of reconstructed volume obtained by nano-CT (1 vx = 63.5 nm) for exposed sample. Voxels denser than threshold value (TV, dotted line) attributed to NMs, are colored in red in Figures 3.D and 4.A.

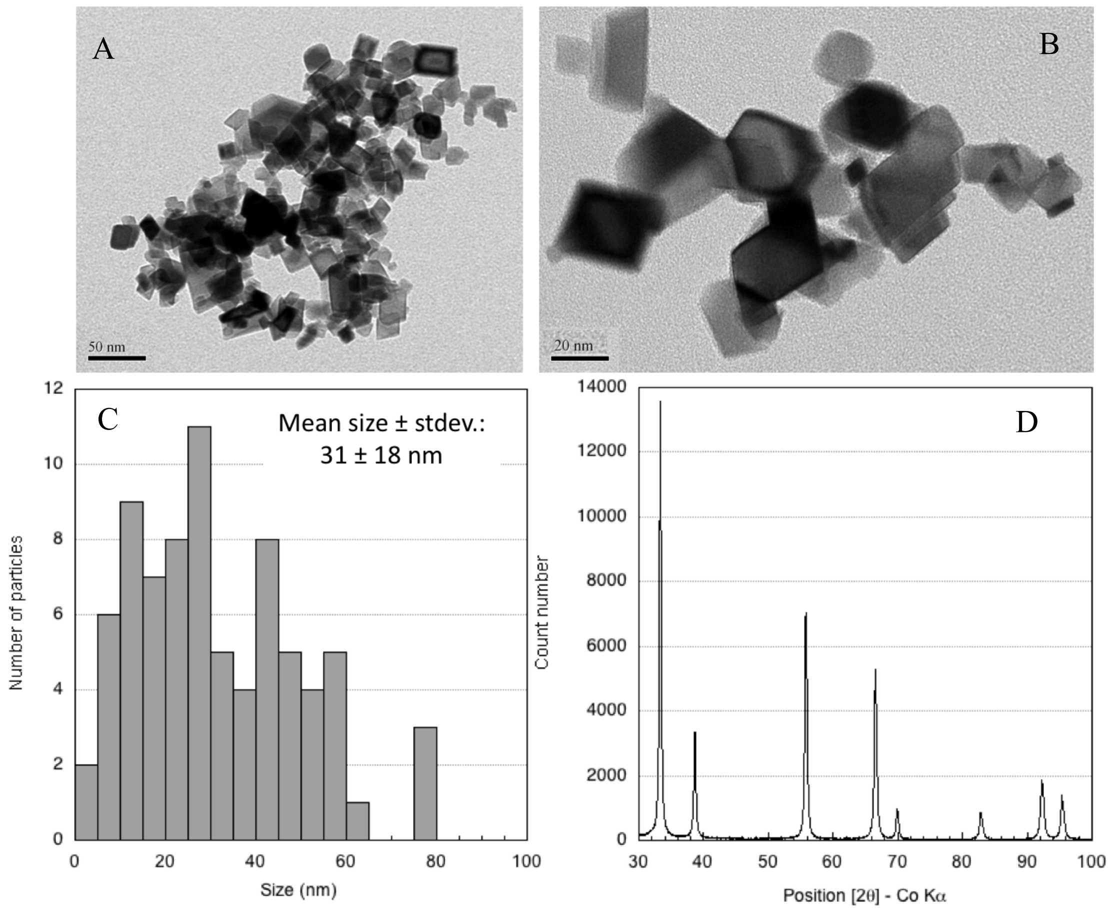

Figure S5: (A, B) representative TEM images of CeO_2-NMs and (C) particle size distribution obtained from statistic image analysis. (D) X-ray diffraction pattern of CeO_2-NMs used (cerianite).

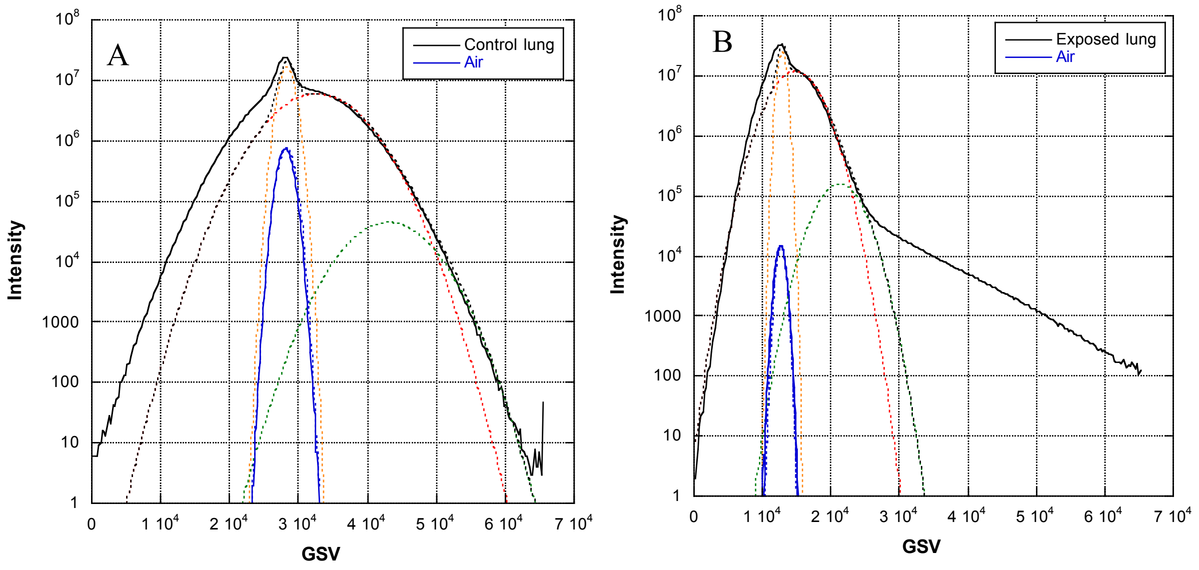

Figure S6: Histograms of reconstructed volumes obtained by micro-CT (HRes scan, 1 vx=1.09 µm). Sub-volumes of (A) control and (B) exposed lung tissue (excluding sample holder, i.e. kapton tube) are considered. First part of lung tissue histograms is well fitted (fit, black dotted line) by the sum of 3 Gaussian functions (blue, orange and green dotted lines). Air sub-volume histograms are well fitted with a Gaussian function (black dotted lines).
